# New Vaccine Design Based on Defective Genomes That Combines Features of Attenuated and Inactivated Vaccines

**DOI:** 10.1371/journal.pone.0010414

**Published:** 2010-04-29

**Authors:** Teresa Rodríguez-Calvo, Samuel Ojosnegros, Marta Sanz-Ramos, Juan García-Arriaza, Cristina Escarmís, Esteban Domingo, Noemí Sevilla

**Affiliations:** 1 Centro de Investigación en Sanidad Animal, Instituto Nacional de Investigación y Tecnología Agraria y Alimentaria (INIA), Madrid, Spain; 2 Centro de Biología Molecular Severo Ochoa (CSIC-UAM), Madrid, Spain; 3 Centro de Investigación Biomédica en Red de Enfermedades Hepáticas y Digestivas (CIBERehd), Barcelona, Spain; Institut Pasteur, France

## Abstract

**Background:**

New vaccine designs are needed to control diseases associated with antigenically variable RNA viruses. Foot-and-mouth disease (FMD) is a highly contagious disease of livestock that inflicts severe economic losses. Although the current whole-virus chemically inactivated vaccine has proven effective, it has led to new outbreaks of FMD because of incomplete inactivation of the virus or the escape of infectious virus from vaccine production premises. We have previously shown that serial passages of FMD virus (FMDV) C-S8c1 at high multiplicity of infection in cell culture resulted in virus populations consisting of defective genomes that are infectious by complementation (termed C-S8p260).

**Principal Finding:**

Here we evaluate the immunogenicity of C-S8p260, first in a mouse model system to establish a proof of principle, and second, in swine, the natural host of FMDV C-S8c1. Mice were completely protected against a lethal challenge with FMDV C-S8c1, after vaccination with a single dose of C-S8p260. Pigs immunized with different C-S8p260 doses and challenged with FMDV C-S8c1 either did not develop any clinical signs or showed delayed and mild disease symptoms. C-S8p260 induced high titers of both FMDV-specific, neutralizing antibodies and activated FMDV-specific T cells in swine, that correlated with solid protection against FMDV.

**Conclusions:**

The defective virus-based vaccine did not produce detectable levels of transmissible FMDV. Therefore, a segmented, replication-competent form of a virus, such as FMDV C-S8p260, can provide the basis of a new generation of attenuated antiviral vaccines with two safety barriers. The design can be extended to any viral pathogen that encodes *trans*-acting gene products, allowing complementation between replication-competent, defective forms.

## Introduction

Application of genetically modified viruses, deficient in replication, that are incapable of developing a productive infection is a promising strategy to design safe and effective vaccines. We have carried out extensive studies on viral population dynamics using an important pathogen in animal health, foot-and-mouth disease virus (FMDV). FMDV is an aphthovirus of the *Picornaviridae* family. Its genome is a positive strand RNA molecule of about 8,500 nucleotides. It encodes a single polyprotein which is post-translationally processed by viral proteases into the structural proteins (VP1–VP4) of the viral capsid, as well as nine different mature non-structural proteins, and several processing intermediates, involved in replication functions [1], [2], [3],[4]. Sequential passages of biological clone FMDV C-S8c1 in BHK-21 cells at high multiplicity of infection (MOI) resulted in the generation and dominance of defective RNAs which complemented each other to produce progeny, and to kill cells in the absence of standard virus [5], [6]. The different defective genomes that were characterized included a deletion of either 417 nucleotides in the L (leader protease)-coding region, or of 999 or 1017 nucleotides within the capsid-coding region. Other deletions were also present at lower frequencies [6]. Because the deletions of 417 and either 999 or 1017 nucleotides were the most frequent, we refer to two classes of defective genomes. However, as for any RNA virus, the population consists of a swarm of non-identical genomes with mutations and internal deletions [4]–[6]. Upon replication and complementation in the same cell, the two classes of defective genomes were efficiently replicated, and were packaged into separate viral particles [5], [6]. Therefore, in following rounds of infection, at least two particles bearing different deletions had to co-infect the same cell for progeny virus production [7]. This defective-complementing virus system was termed C-S8p260. The limitation of C-S8p260 to spread upon dilution should offer a safety barrier against transmission of infectious virus following an initial replication *in vivo*.

FMD is one of the most economically devastating diseases of cloven-hooved animals, that results in great reduction of productivity in adult farm animals, high mortality among young animals, and severe restrictions in the trade of animals and their products [8], [9], [10], [11]. The threat posed by FMDV is due to a combination of virological characteristics such as high contagiousness, virion stability in the environment (including survival in infected carcasses), a rapid replication cycle, short incubation times, and high level of virus excretion via aerosols. Other features that contribute to the difficulties encountered to combat the disease are high mutation rates and the quasispecies nature of viral populations, that lead to antigenic variation, reflected in the seven serotypes and the numerous antigenic variants described to date (reviewed in [12], [13]). Antigenic diversity and heterogeneity of FMDV require the periodic updating of multivalent vaccine formulations to match the antigenic specificity of the vaccine with that of the viruses circulating in the field. FMDV is a global problem since it is endemic in parts of Asia, Africa, the Middle East and South America, with sporadic outbreaks in other areas considered free of the disease such as Europe and Japan. Therefore, controlling both endemic and epidemic FMD has become a global aim.

Currently, vaccination and culling of infected animals and their contacts are the major means to control FMD [3]. Vaccination in endemic areas is implemented mainly by periodic administration of a vaccine based on chemically inactivated virus [14], [15]. Despite extensive research to develop a synthetic vaccine against FMD, no peptide- or protein subunit-based anti-FMD vaccines have been licensed. Virus immunization with conventional vaccines usually elicits high levels of circulating neutralizing antibodies, which, together with a T-cell response, correlate with protection against homologous and antigenically closely related FMDVs [16], [17], [18], [19].

In the present report we evaluate the immunogenicity of C-S8p260 and its capacity to confer protection against challenge with FMDV C-S8c1 using an adult mouse model [20] and swine, the natural host of the parental, virulent FMDV C-S8c1. Specifically, all experiments are being carried out with a mixture of virus variants (collectively called C-S8p260) all of which are harbouring defective genomes (deletions of 417 and either 999 or 1017 nucleotides) that individually are not infectious but by complementation will yield plaque upon co-infection. The rationale for this design was to enhance the effectiveness of presentation of viral antigenic sites, through transient replication of the defective ensemble, capable of stimulating a response to B- and T-cell epitopes. We show that indeed the defective virus population is able of eliciting a cellular and humoral immune response, but not of developing viremia, or being transmitted from vaccinated to contact animals. Thus, we propose a new design of an attenuated vaccine consisting of complementing, defective genomes, thereby incorporating safety features of an inactivated vaccine. The design can be applied to other viral pathogens whose replication tolerates internal deletions of genomic regions whose expression products can be complemented in *trans*.

## Results

### Immunization with C-S8p260 confers protection against FMDV in mice

**Figure 1:**
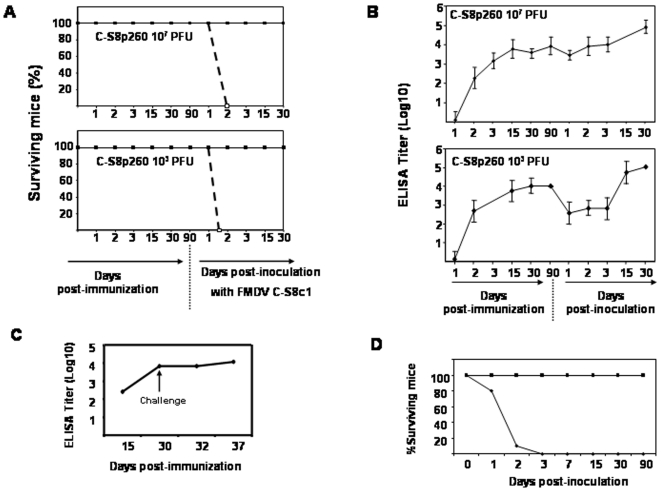
Immunogenicity of C-S8p260 in mice. C57BL/6 mice were inoculated in the FP with either 10^3^ or 10^7^ PFUs of C-S8p260, or PBS. At 90 days post-immunization, mice were challenged with 10^4^ PFUs of FMDV C-S8c1 (procedures are described in Materials and Methods). A. Survival curves after immunization and after challenge (indicated in the abscissa, with arrow in the bottom panel). Continuous line: animals immunized with C-S8p260; discontinuous line: animals inoculated with PBS. B. IgG titers determined by ELISA at different days post-immunization and after challenge (abscissa and bottom panel). C. IgG titers in serum of mice immunized with BEI-inactivated C-S8p260. The day of the challenge with 10^4^ PFUs of C-S8c1 is indicated with an arrow; 16 animals were inoculated in each group. D. Survival of mice inoculated with C-S8p260p3d and C-S8c1; 21 mice were inoculated with 10^7^ PFUs C-S8p260p3d (squares), and 10 mice with 10^3^ PFUs of C-S8c1 (diamonds).

Adult C57BL/6 mice are a valid surrogate model for FMDV infections, with features of pathogenesis shared with natural hosts of FMDV [20], [21]. Previous studies indicated that subcutaneous inoculation at 10^3^ PFUs of C-S8c1 in the footpad (FP) of adult C57BL/6 mice resulted in 100% mortality within 2–3 days [20]. Thus, we used C57BL/6 mice as an animal model to evaluate the immunogenicity and capacity of the defective genome-based population C-S8p260 to confer protection against infection with FMDV C-S8c1. Two groups of sixteen mice were inoculated in the FP with either 10^3^ or 10^7^ PFUs of C-S8p260 (see Materials and Methods for specific determination of C-S8p260 titer); four animals were inoculated with PBS, as control. Each mouse was bled at days 1, 2, 3, 15, 30 and 90 post-inoculation (pi) to determine levels of virus antigen, and antibody titers elicited by C-S8p260. No virus was detected in blood samples at any time pi with either dose of C-S8p260 by plaque assay in BHK cells and FMDV RNA quantification as described in Materials and Methods. In addition, FMDV RNA was not detected in several tissue samples (heart, pancreas, spleen, liver, lung and kidney) from C-S8p260 inoculated mice at 48 hours pi by FMDV RNA quantification (data not shown). The animals showed no signs of disease, indicating that the mixture of C-S8p260 genotypes is highly attenuated for adult C57BL/6 mice. At day 90 pi, mice were challenged with 10^4^ PFUs of FMDV C-S8c1 (ten times the lethal dose [20]), and viremia and survival rates were examined. All the unvaccinated control animals died at day 2 post-challenge (pc), as expected [20]. In contrast, none of the mice vaccinated with C-S8p260 died or developed any symptom associated with FMDV replication (Figure 1A). FMDV-specific IgG levels increased following immunization (Figure 1B). More interesting, significant neutralizing antibody titers were measured in sera from all mice just before challenge, and the titers did not increase following the challenge with FMDV C-S8c1, except for animals # 7, 22 and 27 that had low antibody titers prior to challenge (Table 1). No significant differences in total IgG or in neutralizing antibody titers were found between mice inoculated with 10^3^ PFUs or 10^7^ PFUs of C-S8p260. These data indicate that mice immunized with C-S8p260 were able to elicit an immune response against FMDV which was potent enough to confer protection.

**Table 1:** Neutralizing antibody response against FMDV C-S8c1, in mice, after vaccination with C-S8p260.

C-S8p260^a^	Neutralization titer (PRN70)^b^	
10^7^ PFUs	30 days PI^c^	3 days PC^d^
Mouse #		
1	2.0	1.8
2	2.0	2.0
3	2.4	2.2
4	2.1	1.9
5	1.3	1.5
6	1.7	1.8
7	0.4	1.9
8	1.7	1.8
9	2.7	2.4
10	1.9	1.9
10^3^ PFUs		
Mouse #		
20	2.0	1.9
21	2.1	1.8
22	<0	1.8
23	1.7	1.8
24	1.4	2.4
25	1.6	1.8
26	1.4	1.8
27	0.6	1.1
28	1.4	1.5
29	1.7	1.8

a Mice were vaccinated with 10^7^ PFUs or 10^3^ PFUs of C-S8p260, indicated for each group.

b Neutralization titers are expressed as PRN70 (plaque-reduction neutralization titer 70): the reciprocal of the dilution of serum that causes neutralization of 70% of PFU.

c 30 days post-immunization.

d 3 days post-challenge with 10^4^ PFUs of C-S8c1.

To investigate whether C-S8p260 could be used also as an inactivated vaccine, we have used binary ethylenimine (BEI) inactivation as an effective method to achieve the irreversible loss of viral infectivity with minimal loss of antigenic properties [22], [23], [24]. ELISA test against a panel of monoclonal antibodies specific for the main FMDV antigenic site (site A) have shown no differences in antigen recognition between C-S8p260-BEI-inactivated virus and C-S8p260 (data not shown). Mice were inoculated intramuscularly with BEI-inactivated C-S8p260 (equivalent to 5×10^6^ PFUs, see Materials and Methods for the correction between measured PFUs and number of infectious particles), and received a booster with identical dose and composition at day 15 post-immunization. At 15 days post-booster the animals were challenged with 10^4^ PFUs of C-S8c1 in the FP. Following challenge, immunized mice showed no clinical signs or death associated with FMDV replication, including viremia, while control, non-immunized mice died by day 2, as expected [21] (data not shown). High titers of FMDV-specific IgGs were determined before challenge, and titers did not increase significantly after challenge (Figure 1C). These data suggest that mice vaccinated with BEI-inactivated C-S8p260 elicited a FMDV-specific immune response able to protect mice against a lethal dose of FMDV C-S8c1.

One main concern is the possibility that reconstruction of standard virus by recombination between two defective genomes would generate a competent virus able to replicate and cause disease. To determine the virulence of the standard virus population (C-S8p260p3d), rescued by recombination between the two defective forms [5], [6], 21 mice were inoculated with 10^7^ PFUs of C-S8p260p3d in the FP, the maximal dose that can be obtained with C-S8p260p3d. None of the mice inoculated with C-S8p260p3d died or developed any clinical symptom associated to FMDV replication (Fig. 1D). Thus, the replication-competent virus C-S8p260p3d is highly attenuated for mice (see also Discussion).

### Pigs immunized with C-S8p260 were protected against FMDV C-S8c1 challenge

The effectiveness of C-S8p260 as a potent FMD vaccine in mice prompted us to investigate the efficacy of C-S8p260 as a vaccine in pigs, one of the natural hosts of FMDV, and the host from which FMDV C-S8c1 was isolated [25]. To evaluate C-S8p260 as a vaccine in swine, four different vaccination regimes were used (Table 2). In group 1, six pigs (pigs numbered 1 to 6) were vaccinated with 5×10^6^ PFUs of C-S8p260 with incomplete Freund's adjuvant, and boosted 15 days post-immunization with the same dose and composition of antigen. In group 2, three pigs (numbers 7 to 9) were vaccinated with 10^3^ PFUs of C-S8p260 with adjuvant, and boosted 15 days post-immunization with the same dose and composition of antigen. In group 3, two pigs (numbers 10 and 11) were vaccinated with 5×10^6^ PFUs of C-S8p260 without adjuvant, and boosted 15 days post-immunization with the same amount of antigen, without adjuvant. In group 4, three pigs (numbers 12 to 14) were vaccinated with BEI-inactivated C-S8p260 with adjuvant, and boosted with the same dose and composition of antigen at 15 days post-immunization. Finally, in group 5, 3 pigs (numbers 15 to 17) were administered PBS, and served as negative controls. All groups were challenged at 18 days post-booster (33 days post-immunization) with 10^5^ PFUs of FMDV C-S8c1, and clinical signs were scored for 12 days, as described in Materials and Methods.

**Table 2:** Clinical manifestation in swine inoculated with C-S8p260 and challenged with FMDV C-S8c1.

Group	Inoculum(PFUs of C-S8p260)	Animal	Viremia (dpc, day of onset, duration in days)^a^	Nasal swab specimen (dpc, day of onset, duration [days])^b^	No. of lesions (day of onset)^c^
1	5×10^6^+Adj	1	Neg.	Neg.	Neg.
		2	Neg.	Neg.	Neg.
		3	Neg.	Neg.	Neg.
		4	Neg.	Neg.	Neg.
		5	Neg.	Neg.	Neg.
		6	2.5×10^5^ (4, 4, 1) [Neg.]	Neg.	Neg.
2	10^5^+Adj	7	Neg.	Neg.	Neg.
		8	Neg.	Neg.	Neg.
		9	Neg.	Neg.	Neg.
					
3	5×10^6^	10	2.5× 10^5^ (2, 2, 2) [Neg.]	Neg	Neg
		11	3.1×10^5^ (2, 2, 2) [Neg.]	Neg	Neg
4	BEI inactivated	12	7×10^5^ (2, 2, 4) [1.2×10^4^]	2.8×10^5^ (4, 4, 1)	5 (3)
		13	2×10^5^ (2, 2, 4) [1×10^4^]	3.7×10^6^ (4, 4, 1)	5 (4)
		14	Neg.	Neg.	Neg.
5	Non vaccinated	15	6.2×10^9^ (3, 1, 5) [1×10^5^]	2×10^8^ (4, 2, 2)	15 (2)
		16	7×10^9^ (3, 1, 5) [1.1×10^5^]	1.2×10^7^ (4, 3, 2)	16 (2)
		17	1.1×10^9^ (3, 1, 6) [6.5×10^4^]	1.1×10^6^ (4, 3, 2)	11 (2)

a Viremia is expressed as FMDV RNA molecules/ml of serum quantified by ^q^RT-PCR (Neg. means<10^3^ FMDV RNA molecules/ml of serum). The three numbers given in parenthesis correspond to: dpc, the day after challenge at which the maximum level of viremia was detected; the first dpc at which viremia was detected; and the third value is the number of days of duration of viremia. The infectivity of those sera that were positive for FMDV RNA is indicated in brackets as PFU/ml of serum; [Neg.] means<100 PFU/ml of serum.

b FMDV RNA molecules per ml of nasal secretion (Neg. means<10^3^ FMDV RNA molecules per ml of nasal secretion). The dpc, onset, and duration values are as defined in footnote *a*.

c Number of toes with lesions plus the snout and tongue combined, when lesions were present. The maximum score is 17. The day of onset (given in parenthesis) is the first day after challenge at which lesions were detected.

Animals in the control group (group 5) developed clinical signs of disease by 2 days pc, and had a significant lesion score (Table 2). In contrast, the animals to which C-S8p260 was administered (groups 1, 2 and 3) never developed any clinical signs of disease. Two out of three animals in group 4 (pigs 12 and 13) vaccinated with BEI-inactivated C-S8p260 showed delay in the onset of clinical symptoms, with a low score (5), while pig 14 did not show any clinical symptom. Comparison of the results of groups 1, 2 and 3 suggest that transient replication of C-S8p260 in swine contributed to increase the level of protection (see Discussion).

Viremia was evaluated in all the animals by determination of infectivity (PFUs/ml) and FMDV RNA level in serum (Table 2). The control group (group 5) developed viremia at day 1 pc, lasted for 5–6 days, and reached an average peak value of 1×10^5^ PFUs/ml of serum. The animals in groups 1 and 2 did not develop viremia detected by infectivity (<10 PFU/ml serum). However, the serum of animal 6 in group 1 was positive for FMDV RNA (10^5^ RNA molecules/ml of serum), despite no detectable infectivity. The RNA levels were 10^4^-fold lower than in the control group (1–7×10^9^ RNA molecules/ml serum). All the animals in group 3 and 4 developed viremia detected by real time RT-PCR, but the onset of viremia was delayed, viremia lasted for a shorter period of time, and the number of FMDV RNA molecules was generally 10^3^- to 10^4^-fold lower than in the control animals. One exception was animal 14 (group 4) that did not develop any viremia or clinical symptom. Viral RNA was also detected in the nasal swabs of the control group and in animals of group 4, although the duration of positive FMDV RNA was shorter than in the control group. No virus was detected in the nasal swabs of protected animals. These results indicate that immunization of pigs with C-S8p260 plus adjuvant conferred solid protection against FMDV C-S8c1 challenge, and limited the replication of FMDV in the challenged animals, that did not contain detectable viral RNA in their nasal secretions.

### C-S8p260 induces systemic FMDV-specific antibodies and high titers of neutralizing antibodies

**Figure 2:**
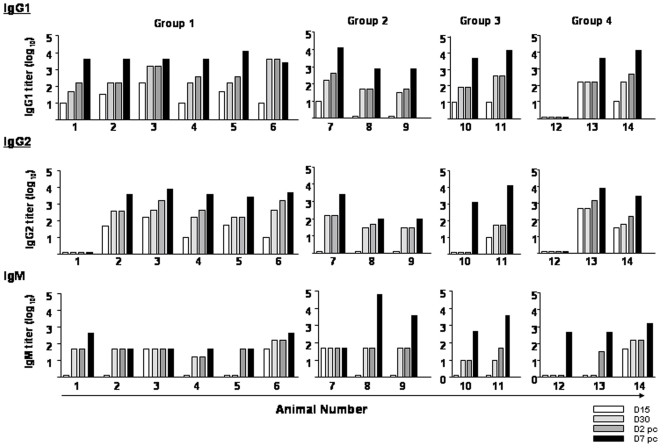
Serum IgG1, IgG2 and IgM-specific responses in pigs following immunization with C-S8p260. Each column corresponds to the antibody titer for the indicated animal (numbers 1 to 14) at different times (D, day) post-immunization and post-challenge (white bars: D15 pi; light gray: D30 pi; dark grey: D32 pi (that corresponds to D2 post-challenge), and black: D37 pi (that corresponds to D7 post-challenge). Titers are expressed as the reciprocal of the highest dilution of serum (log_10_) that gives an OD_A460_ of twice the value obtained with the pre-immune serum of the corresponding animal.

FMDV-specific antibody responses were quantitated by ELISA in serum of pigs inoculated with C-S8p260. IgG levels systematically rose from those scored at 2 days post-challenge (dpc) to those scored at 7 dpc (Figure 2). The only animal that did not develop any FMDV-specific antibody was number 12 of group 4, and this animal was not protected against FMDV challenge (Table 2). IgG1 levels were only slightly higher than IgG2 responses. Exceptions were animals 1 and 10 of groups 1 and 3, respectively. Animal 1 did not develop any detectable IgG2 at any time, and animal 10 did not produce IgG2 until 7 days pc. Vaccination also resulted in a rapid IgM response. In most of the animals (except numbers 2, 3 and 7), there was an increase of the IgM responses by day 7 pc.

FMDV-specific neutralizing antibodies were found in sera from all pigs after vaccination, except in pig number 12 (Table 3). All animals in group 1, and animals 7, 13 and 14 showed significant neutralization titers (value of PRN_70_>1.2) after the first vaccine administration. After booster vaccination, titers increased in all animals, even in those that did not display significant neutralization titer after administration of the first dose. Neutralization titers increased slightly at 2 and 7 days pc.

**Table 3:** Neutralizing antibody response to FMDV C-S8c1 in sera of pigs vaccinated with C-S8p260.

Animals	Virus neutralization titers (VNT)^a^ at the indicated day after immunization				
	0	15	30	32 (D2 pc)^b^	39 (D7 pc)^c^
Group #1					
1	<0	1.2	1.8	2.5	3.7
2	<0	1.3	1.5	3.0	3.6
3	<0	2.2	3.4	3.4	3.5
4	<0	1.9	2.5	2.5	3.3
5	<0	1.5	2.3	2.3	3.4
6	<0	1.3	2.8	2.7	2.7
Group #2					
7	<0	2.4	2.3	2.5	2.8
8	<0	<0	1.8	2.3	3.0
9	<0	<0	2.2	2.5	3.0
Group #3					
10	<0	<0	1.8	1.8	2.7
11	<0	<0	1.4	1.3	3.0
Group #4					
12	<0	<0	<0	1.7	1.7
13	<0	0.4	2.0	2.0	3.1
14	<0	2.5	2.5	2.6	3.2
Group #5					
15	<0	<0	<0	<0	2.3^d^
16	<0	<0	<0	<0	3.1^d^
17	<0	<0	<0	<0	2.2^d^

a VNT, virus neutralization titer (log_10_ of the reciprocal of the last dilution able to neutralize 70% of 100 PFUs of FMDV C-S8c1).

b Day 2 post-challenge with 10^5^ PFUs of FMDV C-S8c1.

c Day 7 post-challenge with 10^5^ PFUs of FMDV C-S8c1.

d For control animals, VNT was obtained at day 12 pc.

**Figure 3:**
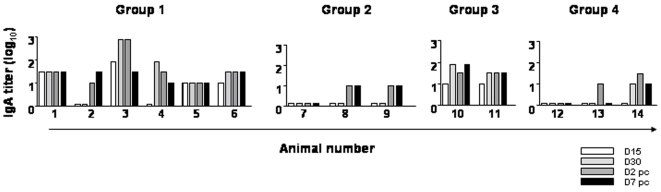
Serum IgA-specific responses in pigs following immunization with C-S8p260. Each column corresponds to the antibody titer for the indicated animal (numbers 1 to 14) at different times (D, days) post-immunization and post-challenge (white bars: D15 pi; light gray: D30 pi; dark grey: D32 pi (that corresponds to D2 post-challenge), and black: D37 pi (that corresponds to D7 post-challenge). Titers are expressed as the reciprocal of the highest dilution of serum (log_10_) that gives an OD_A460_ of twice the value obtained with the pre-immune serum of the corresponding animal.

The effect of vaccination in systemic IgA responses was also evaluated due to their role in mucosal immunity and their relevance for protection against FMDV [26], [27]. Animals in group 1 and 3 showed a strong induction of IgA early after vaccination with the exception of animal 2 (Figure 3). No IgA induction was observed in other groups. This suggests that a high dose of C-S8p260 might be required to induce a specific IgA response that may contribute to FMDV mucosal immunity. Taken all these data together, we conclude that the vaccine formulation based on replication-competent defective FMDV genomes is able to evoke a FMDV-specific humoral immune response in swine.

### C-S8p260 induces a FMDV-specific T cell response

**Figure 4:**
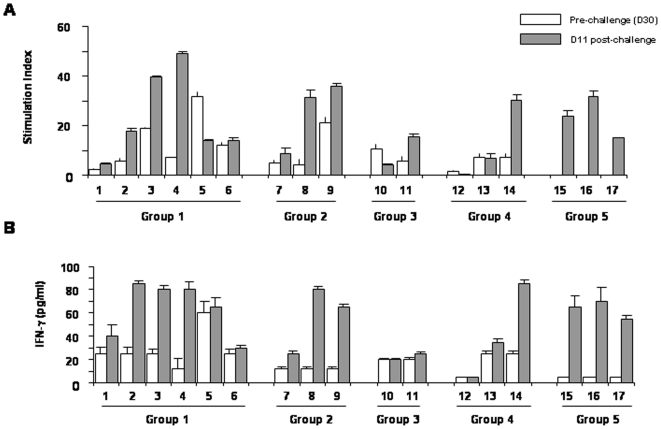
FMDV-specific T cell responses in vaccinated pigs. PBMCs were purified from blood of animals at day 30 (D30) post-immunization (pre-challenge) (white bars), and D11 post-challenge (grey bars). A. Lymphoproliferative responses of pigs 1 to 17 (groups 1, 2, 3, 4 and 5) to FMDV C-S8c1. Data are shown as stimulation index (see Material and Methods) and standard deviations are indicated. B. IFN-γ release by PBMC stimulated with FMDV C-S8c1. Values were determined at 72 h of in vitro stimulation. The detection level in control cultures (medium alone) were below the sensitivity of the assay (5 pg/ml).

To evaluate the induction of FMDV-specific T cells as a result of vaccination, lymphoproliferation assays were carried out with PBMCs of vaccinated pigs. The stimulation indexes (SI) of PBMCs at day 30 post-vaccination (pre-challenge) and day 11 post-challenge were compared (Figure 4A). Highly specific responses (SI>6) were detected in all groups, except in animal 12 of group 4. Proliferative responses were also detected after challenge, with high values of SI for animals 2, 3, 4, 8, 9, 11 and 14. By contrast, animals 1, 5, 6, 7, 10 and 13 did not show any significant increase in the SI after challenge, with animals 5 and 10 showing lower values of SI after challenge than before challenge. The control group (group 5) showed a significant SI at day 11 pc. In addition, we have determined the production of IFN-γ in supernatants of PBMCs from vaccinated pigs at day 30 post-vaccination (pre-challenge) and day 11 pc. In both cases, the IFN-γ levels correlated with the lymphoproliferative responses (Figure 4B). This pattern of cytokine production and the lymphoproliferative responses indicates that immunization with C-S8p260 primes T cells that can recognize the viral epitopes presented in the context of a subsequent viral encounter. Thus, a vaccine consisting of defective, complementing versions of the FMDV genome can evoke both an antibody and a cellular immune response, which results in protection of swine when challenged with the corresponding virulent FMDV.

## Discussion

The work presented here reports a potential use as vaccine of unique viral particles that we discovered upon extensive passage of FMDV in BHK-21 cells [5]. We show that defective genomes that can replicate in the absence of wild type virus confer full protection in mice and pigs against FMDV challenge. Such a bipartite particle system (with two classes of genomes with internal deletions, but consisting of a mutant spectrum, with multiple minority genomes with non-identical internal deletions [6]) can be regarded as a candidate anti-FMD vaccine. Its key features are that the initial virus replication in the animal would mimic a true live-attenuated virus, and that subsequent rounds of infection would be limited by the requirement of co-infection of the cells by two different classes of viral particles. The results cannot exclude that at the site of inoculation a full length recombinant genome could be generated, and that its transient replication could contribute to the observed immune response. However, if reconstruction of standard virus by recombination occurred in the vaccinated animals, it would generate a replication-competent but highly attenuated FMDV recombinant (Figure 1D), a result expected from previous results of attenuation for mice and cattle of FMDV passaged in cell culture [28], [29] (Rodríguez-Calvo and Sevilla, unpublished results). Thus, it is highly unlikely that a recombinant standard virus could arise in the animals and acquire the mutations needed for virulence. This is because the segmented form has manifested a selective advantage relative to the corresponding standard virus generated by recombination (48; Ojosnegros et al., manuscript in preparation). Since generation of the recombinant necessitates co-infection of the same cell by the two types of defective genomes it is unlikely that the recombinant would gain in the competition. Under these conditions it seems unlikely that the recombinant, standard genome, would undergo sufficient rounds of replication to acquire the virulence-associated mutations. In fact such acquisition would be less likely than upon administration of the standard genome alone. As documented in Results, no virulent variants were detected upon administration of the standard, recombinant-derivative genome in isolation.

Classic whole-virus inactivated vaccines against FMD have been successful in controlling the disease, but have met with the problem of outbreaks associated either with incomplete inactivation of the virus during vaccine manufacturing or with escape of virus from vaccine production factories [30]. The development of new, safe and effective vaccines to control FMD must take into consideration the problem of the extensive antigenic heterogeneity of natural FMDV populations, including antigenic differences among viruses of the same disease outbreak [31], [32], [33]. Antigenic heterogeneity is a consequence of high mutation rates and quasispecies dynamics, and it is common to most pathogenic RNA viruses (reviewed in [34]). For a vaccine to be effective to control highly variable RNA viruses, it must evoke a broad immune response targeted to many independent T-cell and B-cell epitopes, in order to prevent selection of vaccine-escape mutants. Despite a broad immune response, the vaccine is unlikely to confer protection against infection by viruses of a different serotype, and multivalent vaccines are required. Such a broad immune response can, in principle, be best accomplished with live-attenuated virus vaccines, and it is unlikely to be achieved with individual surface proteins expressed in heterologous vector systems, or with synthetic peptides that represent a restricted number of epitopes. These limitations have been amply manifested in previous unsuccessful attempts to produce a synthetic vaccine against FMD [35], [36], [37], [38], [39].

Live-attenuated viral vaccines that replicate in the host without producing disease expose the host to a greater antigenic mass and diversity of epitopes than the corresponding whole-virus inactivated vaccine. In addition, if delivered by the natural route, attenuated vaccines can induce mucosal immunity [40]. However, this basic principle of vaccinology is not easy to put into practice for several reasons. One is that, because of the error-prone replication of RNA viruses, attenuated virus variants can revert to virulent forms, as extensively documented for live poliovirus vaccines [41], [42], [43]. While attenuated anti-FMD vaccines were used in some countries [10], [44], they were replaced by chemically inactivated whole virus antigen used in conjunction with an adjuvant, for safety considerations. Nevertheless, new promising versions of live-attenuated anti-FMDV vaccines have been developed. Thus, genetically engineered FMDV serotype A12 lacking the coding region for the L proteinase induces a partial protective immune response in cattle and swine against FMDV challenge [45], [46]. However, attenuated vaccines engineered to have a large genetic barrier to reversion may produce too weak an immune response (excess attenuation), resulting in low effectiveness.

These multiple problems led us to investigate the use of C-S8p260, a bipartite FMDV version of infectious particles, as a potential FMDV vaccine. The original passage experiments of FMDV C-S8c1 in BHK-21 cells were undertaken to determine a possible limit for fitness gain of a virus replicating in a constant biological environment, one of the open questions in viral population dynamics [47]. Unexpectedly, the virus evolved towards genomic forms with internal deletions that were packaged into separate particles and that required complementation to produce infectious progeny and kill cells [5],[7]. Both live C-S8p260 and BEI-inactivated C-S8p260 conferred protection to C57BL/6 mice, but sterile protection of swine required live C-S8p260 (Table 2). This suggests a role of viral replication in the induction of a protective response. Since viremia was absent in mice and swine vaccinated with C-S8p260, replication of C-S8p260 (or a possible recombinant with a full length genome) must have been transient and probably limited to the inoculation site and surrounding tissues. Absence of viremia is consistent with the requirement of C-S8p260 progeny to co-infect cells to sustain the infection. Dilution of progeny particles (into surrounding tissues, blood) should effectively interrupt the progress of the infection [7]. It may be argued that the observed benefits of a vaccine based on C-S8p260 over the corresponding BEI-inactivated vaccine were not due to a transient replication of the live C-S8p260 but to loss of epitopes as a result of the BEI inactivation. Although it cannot be excluded that some epitopes might be lost as a result of treatment of C-Sp260 with BEI, previous investigations as vaccine preparation and efficacy led to the choice of BEI as an adequate reagent to inactivate FMDV while maintaining its immunogenic properties [23]. Furthermore, we have compared the antigenic properties of C-S8p260 and C-S8p260-BEI-inactivated and did not show any difference (data not shown). Concerning an initial replication of C-S8p260, it would be technically very difficult to prove or disprove its occurrence. At least from our experimentally observations in cell culture [5], [6], [48] and theoretical studies on bipartite virus dispersal [7] it is extremely unlikely that the cell adjacent to the inoculation site would not be dually infected by the two particle components of C-S8p260. Yet, it is not possible to anticipate how many rounds of multiplication of C-S8p260 will be established in cells progressively distant from the inoculation site because finally viral dilution will stop progress of the infection [7], as evidence by the absence of viremia. Independently of the number of rounds of progeny production, a vaccine based on two-component viral preparation will have a safety barrier additional to mere attenuation of a standard one-component virus, and immunogenicity may still be maintained upon chemical inactivation.

The solid protection observed by C-S8p260 correlated with the detection of high levels of circulating neutralizing FMDV antibodies. Interestingly, FMDV-specific IgA antibodies were detected mainly in animals from group 1 and 3, those vaccinated with the highest antigen dose (5×10^6^ PFUs of C-S8p260) (Figure 3). The induction of IgA response upon vaccination with C-S8p260, and its possible role in protection against challenge with FMDV, merits further investigation. Vaccination with C-S8p260 also elicited T cells that consistently proliferated when stimulated with FMDV (Figure 4). After FMDV challenge, proliferation was maintained or increased, suggesting that C-S8p260 primes T cells that can recognize the viral epitopes presented in the context of a subsequent virus encounter. Altogether, our results suggest that vaccination with C-S8p260 induces T- and B-cell activation that efficiently contributes to FMDV protection. Obviously, protection is not expected to extend to other FMDVs of a different serotype. Additional work is needed to investigate the response of vaccinated animals upon challenge with heterologous virus, in particular other FMDV subtypes within type C, and also to establish the duration of protection against challenge with homologous virus.

Thus, we arrive at a vaccine based on defective FMDV genomes that recapitulates features of an attenuated (due to the initial viral replication) and of an inactivated (due to low probability of double infections as the virus spreads in the animal) vaccine. The vaccine could be easily marked by a tagged antigen inserted in-frame in the place of the deletion of the defective genomes, a requisite to distinguish infected from vaccinated animals [15]. The new vaccine design encompasses at least two safety levels that apply both to vaccine manufacture and to vaccine administration to animals. The first safety level resides in genome segmentation. Infection (in the vaccinated animals or in the event of virus escape from a production unit) will be self-limiting, as documented by absence of viremia and virus in nasal secretions upon vaccination of swine with C-S8p260. Second, if standard virus is reconstructed from the defective virus, its attenuation together with the evoked immune response, will render disease by such recombinant extremely unlikely. Additionally, as argued in more detail earlier, since defective FMDV particles interfere with replication of the standard virus in cell culture [48], a dominance of the segmented defective FMDV virus over a possible standard virus generated by recombination is expected, and renders very unlikely and reversion of standard virus to a virulent form. Finally, the results reported in our study suggest that higher doses of BEI-inactivated C-S8p260 are also likely to confer protection. If substantiated, this would provide the vaccine with an additional, third safety barrier because it would consist of an inactivated vaccine (as those currently in use [49]) devoid of any potential danger in the event of incomplete inactivation. Experiments to test these possibilities are in progress.

The proposed vaccine could be administered together with interferon, or other immunostimulatory molecules, to evoke short-term protection, as documented for other anti-FMD vaccines [50], [51]. Genomes with internal deletions of the type present in C-S8p260 should not be difficult to construct with other FMDV serotypes, given the similarity in replication cycles of all FMDVs, and the availability of FMDV replicons lacking substantial portions of the genome [52]. Also, the new vaccine design should be applicable to other RNA viruses, since virus versions with additional genomic segments have been engineered for measles virus [53], [54], [55], [56] and for lymphocytic choriomeningitis virus [57]. The genetic flexibility of RNA viruses may be exploited to engineer vaccine strains that combine features of an attenuated and of an inactivated vaccine, to procure an effective protection.

## Materials and Methods

### Ethics Statement

All the experiments involving animals were approved by the ethical review committee at the Centro de Investigación en Sanidad Animal (CISA-INIA), following guidelines set forth the European Union (Directive 86/609/EEC).

### Virus

FMDV C-S8c1 is a plaque-purified virus of the European serotype C, natural isolate C_1_ Santa-Pau Spain 70 [25]. FMDV C-S8p260 is a viral population obtained after 260 serial cytolytic passages of C-S8c1 at high multiplicity of infection in BHK-21 cells, and C-S8p260p3d is a viral population obtained after 3 serial cytolytic passages of C-S8p260 at low multiplicity of infection in BHK-21 cells [5]. The titers given for C-S8p260 through the text are corrected infectivity. Since a PFU requires co-infection of a cell by at least one of each type of particles harbouring different internal deletions in the RNA [5], [6], [25], [48], a correction is needed to convert the observed PFUs into number of infectious particles (n). This correction can be done by applying the formula: n = √4N (PFU), in which N is the total number of cells [7].

### Immunization and experimental infections

C57BL/6 female mice (7–8 weeks old) were used for mice experiments. Mice were given a single dose subcutaneously in the footpad (FP) of 10^7^ or 10^3^ PFUs of C-S8p260. After 90 days post-immunization, mice were challenged with 10^4^ PFUs of C-S8c1 in the FP. For the experiments with swine, 17 domestic Landrace X Large White pigs, 2 months old (approximately 30 to 40 Kg) were divided into five groups: group 1 included 6 pigs that were immunized with 5×10^6^ PFUs of C-S8p260 with incomplete Freud's adjuvant, with a booster immunization 15 days later; group 2 included 3 pigs that were immunized with 10^5^ PFUs of C-S8p260 with incomplete Freud's adjuvant, with a booster immunization 15 days later; group 3 included 2 pigs that were immunized with 5×10^6^ PFUs of C-S8p260 without adjuvant, with a booster immunization 15 days later; group 4 included 3 pigs that were immunized with BEI-inactivated FMDV C-S8p260 (equivalent to 5×10^6^ PFUs) with incomplete Freud's adjuvant, with a booster immunization 15 days later; and group 5 included 3 non-vaccinated pigs as controls. Animals were immunized via intramuscular injection. All animals were challenged at day 30 post-immunization by injection of 10^5^ PFUs of FMDV C-S8c1 in the coronary band of the left rear foot. This route of challenge in swine is one recommended by the OIE. The animals were monitored for 12 days after challenge. Rectal temperatures, lesion data, and the physical conditions of the animals were determined daily. Blood and nasal swab specimens were collected daily for the first 7 days after challenge, and serum samples were collected every other day. Lesion scores of the animals were determined by evaluating the number of digits with lesions and adding the snout and tongue combined, if vesicles were present (maximum score, 17). The animal experiment was performed in the secure disease agent isolation facilities at the Centro de Investigación en Sanidad Animal (CISA-INIA) according to a protocol approved by the Institutional Animal Care and Use Committee.

### Detection of isotype-specific FMDV-antibodies by ELISA

ELISA was used to measure isotype-specific FMDV-antibodies as previously described [58]. Briefly, 96 wells polyvinylchloride (PVC) microplates (Flow Laboratories) were coated at 4°C overnight with rabbit anti-FMDV serotype C. The antigen (FMDV C-S8c1) was added and incubated for 1 hour. Then, sera from mice or pigs were diluted into PBS/3% ovalbumin and analyzed for the presence of isotype-specific antibodies. Monoclonal antibodies specific for swine IgG1, IgG2, IgM and IgA were supplied by Serotec, and mouse IgG by BioRad. Antibody titers were expressed as the reciprocal of the highest dilution giving an OD_A460_ of twice the value obtained with pre-immune serum for each animal.

### Seroneutralization assay

Neutralization of infectivity by swine or mice sera was carried out by plaque-reduction neutralization assay as previously described [59]. Briefly, FMDV C-S8c1 (100 to 200 PFUs) was incubated with serial dilutions of serum for 1 hour at 25°C, at RT. Then the mixtures were plated on BHK-21 cell monolayers, and plaques visualized by crystal violet staining. Neutralization titers are expressed as the reciprocal of the serum dilution that causes neutralization of 70% of PFUs (PRN70).

### Lymphoproliferation assay

Proliferation assays were carried out in 96-well flat-bottomed microtitre plates. Cells were plated at a concentration of 4×10^6^/ml, 100 µl/well in RPMI/10% (v/v) fetal bovine serum, and 0.05 mM 2-mercaptoethanol. Triplicate cultures were stimulated with 3×10^6^ PFUs of FMDV C-S8c1. Positive controls of the assay were stimulated with Concanavalin A (Con A) (Sigma, St. Louis, MO) at a final concentration of 1 µg/ml. The cultures were incubated for 3 days at 37°C, 5% (v/v) CO_2_. They were then pulsed overnight with ^3^H thymidine (Amersham, Pharmacia) (0.5 µCi/well). The cells were harvested onto filter mats using a cell harvester, and the incorporation of radioactivity into DNA was measured by liquid scintillation in a Microbeta counter (Pharmacia, Uppsala, Sweden). Data are expressed as stimulation index (SI), calculated by dividing the mean cpm obtained in the stimulated cultures by the mean cpm obtained in the control cultures.

### Cytokine detection

PBMC were incubated with 3×10^6^ PFUs of FMDV C-S8c1, and at 72 h post-incubation supernatants were harvested and analyzed by cytokine production using pig IFN-γ ELISA kit (Bender MedSystems). In each assay, the corresponding cytokine was diluted over the detection range recommended by the manufacturer to generate a standard curve from which sample concentrations (pg/ml) were calculated.

### Viral RNA quantification

RNA was extracted from sera or nasal swabs from infected animals by treatment with Trizol (Invitrogen) according to the instructions of the manufacturer [60]. FMDV RNA quantification was performed by real time RT-PCR using the LightCycler instrument (Roche, Indianapolis, IN, USA) and the RNA Master SYBR green I kit (Roche), as specified by the manufacturer. Quantification was relative to a standard curve obtained with known amounts of FMDV C-S8c1 RNA, using a procedure that has been previously described [5], [61].

### Statistical analyses

Data handling, analyses and graphic representation was performed using Prism 2.01 (GraphPad Software Inc.). Statistic differences were determined using a Student *t* test or a one-way ANOVA (p<0.05).
